# Management of a hospital-wide vancomycin-resistant *Enterococcus faecium* outbreak in a Dutch general hospital, 2014–2017: successful control using a restrictive screening strategy

**DOI:** 10.1186/s13756-021-00906-x

**Published:** 2021-02-18

**Authors:** Veronica Weterings, Anita van Oosten, Ellen Nieuwkoop, Jolande Nelson, Andreas Voss, Bas Wintermans, Joris van Lieshout, Jan Kluytmans, Jacobien Veenemans

**Affiliations:** 1 Department of Infection Control, Amphia Hospital, P.O. Box 90158, 4800 RK Breda, The Netherlands; 2 Department of Infection Control, Admiraal De Ruyter Hospital, P.O. Box 15, 4460 AA Goes, The Netherlands; 3 Department of Infection Control, Elisabeth-TweeSteden Hospital, P.O. Box 90151, 5000 LC Tilburg, The Netherlands; 4 Department of Medical Microbiology, Radboud University Medical Centre, P.O. Box 9101, 6500 HB Nijmegen, The Netherlands; 5 Microvida Laboratory for Microbiology, Amphia Hospital, P.O. Box 90158, 4800 RK Breda, The Netherlands; 6 Julius Center for Health Sciences and Primary Care, UMC Utrecht, Utrecht University, P.O. Box 85500, 3508 GA Utrecht, the Netherlands; 7 Laboratory for Microbiology, Admiraal De Ruyter Hospital, P.O. Box 15, 4460 AA Goes, The Netherlands

**Keywords:** VRE, Vancomycin-resistant enterococci, Resistance, Outbreak, Screening strategy, Isolation strategy

## Abstract

**Background:**

The emergence of vancomycin resistant enterococci poses a major problem in healthcare settings. Here we describe a hospital-wide outbreak of vancomycin-resistant *Enterococcus faecium* in a general hospital in The Netherlands in the period December 2014–February 2017. Due to late detection of the outbreak, a large cohort of approximately 25,000 (discharged) patients was classified as ‘VRE suspected’. Hereupon a mitigated screening and isolation policy, as compared with the national guideline, was implemented to control the outbreak.

**Methods:**

After the outbreak was identified, a screening policy consisting of a single rectal swab culture (with enrichment broth) to discontinue isolation and removing ‘VRE suspected’ label in the electronic patient files for readmitted VRE suspected patients, was implemented. In addition to the on admission screening, periodic hospital-wide point prevalence screening, measures to improve compliance with standard infection control precautions and enhanced environmental cleaning were implemented to control the outbreak.

**Results:**

Between September 2014 and February 2017, 140 patients were identified to be colonised by *vanA* mediated vancomycin-resistant *Enterococcus faecium* (VRE*fm*). Two of these patients developed bacteraemia. AFLP typing showed that the outbreak was caused by a single clone. Extensive environmental contamination was found in multiple wards. Within nine months after the detection of the outbreak no new VRE cases were detected.

**Conclusion:**

We implemented a control strategy based on targeted screening and isolation in combination with implementation of general precautions and environmental cleaning. The strategy was less stringent than the Dutch national guideline for VRE control. This strategy successfully controlled the outbreak, while it was associated with a reduction in the number of isolation days and the number of cultures taken.

**Supplementary information:**

The online version contains supplementary material available at 10.1186/s13756-021-00906-x.

## Background

In line with the increased global spread of multi-drug resistant microorganisms, the prevalence of vancomycin resistance among enterococci is rising. The European Antimicrobial Resistance Surveillance Network (EARS-Net) reported a significant increase of number of vancomycin-resistant *Enterococcus faecium* in invasive isolates from 2015 (10.5%) to 2018 (17.3%) in Europe [1]. In the Netherlands, however, the proportion of VRE in infection-related isolates remained stable and slightly higher than 1% over the past 5 years [2].

Despite this low and stable prevalence, there has been an increase in the number of VRE outbreaks. Whereas vancomycin-resistant enterococci (VRE) were detected only sporadically in the Netherlands before 2012, an increasing number of VRE outbreaks have required considerable time and resources to contain over the past 8 years [2]. In 2018 and 2019, VRE outbreaks were among the largest and the most frequently reported in the Netherlands (period 2018–June 2019: VRE 682 patients in 24 outbreaks versus MRSA 93 patients in 20 outbreaks) [3].

Due to the low virulence of VRE and its ability to survive on hospital environmental surfaces, VRE outbreaks have the potential to become substantial in size before they are detected by routine clinical cultures. Strategies to prevent VRE transmission include screening of contact patients and isolation precautions of (suspected) VRE carriers [4, 5]. The Netherlands has a national control strategy of highly resistant micro-organisms including VRE [6]. During outbreaks, contact patients (those admitted to the same room or ward as the index patient) are pre-emptively isolated while awaiting test results to prevent further spread. In this context, there is ongoing discussion about the number of rectal swabs to be tested—with culture considered as gold standard—before a VRE suspected patient can be declared VRE negative. The negative predictive value of 1 negative rectal swab is considered insufficient, and the Dutch national guideline advocates taking 3–5 rectal cultures on separate days [6]. The combination of late outbreak detection and multiple cultures per contact-patient before VRE carriage can be excluded can result in very large numbers of patients to be isolated and screened, hence it is a substantial burden for hospital infection control departments, leading to significant laboratory costs, and exhaust hospital isolation facilities.

This paper describes an outbreak of VRE*fm* in a general hospital in The Netherlands that was detected in December 2015, following an alert from a neighbouring hospital where VRE*fm* carriage was detected in four recently transferred patients. A hospital-wide screening of all patients admitted for more than 48 h for VRE carriage indicated the spread of VRE among patients admitted to various departments at one of two hospital locations. The outbreak was successfully controlled slightly more than a year after its detection (February 2017).

This outbreak report provides an overview of the interventions that were implemented to control the outbreak, including a screening strategy that was more restrictive than the Dutch national guideline recommends.

## Methods

### Design

We retrospectively describe the interventions that were implemented to control an outbreak of VRE*fm* that occurred between September 2014 and February 2017 in a 364-bed general hospital in a non-endemic setting in in the South West of the Netherlands.

### Setting

The Admiraal De Ruyter Hospital (ADRZ) has approximately 25,000 admissions per year at two different locations (Goes and Vlissingen), and a catchment area of 248,000 inhabitants, and supplies 85–90% of the requested hospital care in this area. Tertiary care patients are referred to the surrounding academic centres. Most patients are admitted to an acute admission unit (AAU), consisting of six single rooms and eight multi-bed room with five beds each (total 46 beds), before being transferred to their specific wards (on average after 48 h).

VRE bloodstream infections are very rare in the hospital: in the past 6 years no VRE bloodstream infections have been detected outside the outbreak period (Additional file 1: Materials S1). The antibiotic consumption rate in the hospital is relatively stable and low over the years: in 2016, the overall antibiotic use was 75.2 defined daily doses (DDD) per 100 patient days.

The internal infection control protocol for VRE was based on the Dutch national guideline for (targeted) screening and control measures [6]. This included contact isolation for VRE-positive patients; pre-emptive isolation and screening for patients with previous admission to foreign healthcare facilities and screening of roommates following an unexpected case of VRE. After discharge of a VRE positive patient, routine cleaning and disinfection of patient rooms with 250 ppm chlorine was performed.

### Participants

Participants were all patients admitted to the Admiraal De Ruyter Hospital (ADRZ) between September 1, 2014 and February 5, 2017. Patients were categorised into three groups according to their VRE status and potential risk of VRE carriage: (1) VRE carrier: VRE-positive patients; (2) VRE suspected patients: all patients with prior hospitalisation in the ADRZ hospital location Goes from September 1, 2014. In the beginning of the outbreak it was unclear whether the outbreak included neighbouring nursing homes or rehabilitation centres, therefore patients transferred from these institutions were also categorised as VRE suspected in the first phase of the outbreak.

Patients with no prior hospitalisation in the ADRZ hospital in the outbreak period, nursing home or rehabilitation centre were categorised as [3] low-risk patients.

### Interventions

Upon detection of the outbreak in December 2015, an outbreak management team was initiated consisting of a clinical microbiologist, an infection control specialist, representatives of the hospital management and of the medical staff, the manager of housekeeping and logistics, and a member of the communication department.

The control measures can be divided into three phases, accompanied by the different screening and isolation policies. Table 1 summarizes the dates, isolation and screening policies per phase and risk group. In Table 2, an overview of the implemented control measures during the outbreak is shown.

**Table 1:** Overview of dates, isolation and screening policies per phase and risk group during VREfm outbreak period

Risk group	Definition	Phase 1 Dec 14, 2015–Mar 20, 2016		Phase 2 Mar 21, 2016–Nov 13, 2016		Phase 3 Nov 14, 2016–Feb 5, 2017	
		Identification strategy	Contact precautions	Identification strategy	Contact precautions	Identification strategy	Contactprecautions
VRE carrier	Patient infected or colonised with VRE*fm*	N/A	Yes^a^	N/A	Yes^a^	N/A	Yes^a^
VRE suspected	All patients with prior hospitalisation in the ADRZ hospital location Goes from September 1, 2014 until start of zero transmission period	Single rectal swab on admission	Yes, while awaiting result of single admission screening	Single rectal swab on admission	No	Single rectal swab on admission	No
	Patients transferred from nursing homes or rehabilitation centres	Single rectal swab on admission	Yes, while awaiting result of single admission screening	Single rectal swab on admission	No	No	No
	Roommate of unexpected VRE carrier	N/A	Yes^c^	3 separate rectal cultures (collection at day 3, 5 and 7 after the last exposure)	Yes^a^	3 separate rectal cultures (collection at day 3, 5 and 7 after the last exposure)	Yes^a^
All patients (incl. low risk)	Hospital wide surveillance	Weekly screening of all patients hospitalised > 2 days^b^	N/A	Weekly screening of all patients hospitalised > 2 days	N/A	Monthly screening of all patients hospitalised > 2 days	N/A

^a^Single room or cohorting with other VRE carriers if insufficient availability of single rooms

^b^In high risk departments (dialysis and ICU) all admitted patients are screened, regardless of the admission time

^c^After cleaning of the ward was completed, patients were separated int two cohorts: clean (new admissions not fulfilling the criteria of ‘VRE suspected’), and VRE suspected, each with dedicated staff. Contact precaution (CP) policy: Single room or—if single room was not available—cohort CP of patients with same VRE status on multi occupancy room. Healthcare workers wear gown with long sleeves and gloves before each contact with the patient or the patient environment. Rooms were daily cleaned, medical devices were cleaning and disinfected (alcohol 70%) before leaving the room, and after patient discharge, the room is cleaned and disinfected (250 ppm chlorine)

**Table 2:** Overview of the implemented control measures during VREfm outbreak period

Overview of infection control measures during outbreak		
2015	December (Phase I)	Detection of the outbreak Single hospital-wide screening limited to patients admitted for at least 48 h
2016	January	Initiation of Outbreak Management Team (OMT)
		Reporting outbreak to the national Early warning and response meeting of Hospital-acquired Infections and AntiMicrobial Resistance (SO-ZI/AMR)
		Electronic labelling of VRE-positive patients (confirmed label) and patients with prior hospitalisation in het ADRZ hospital in from September 1, 2014, or prior hospitalisation in a nursing home or rehabilitation centre (‘VRE suspected’ label)
		Informing local hospital personnel and patients, and surrounding hospitals and nursing homes
		Introducing screening for VRE carriage on admission for all patients with electronic ‘VRE suspected’ label and patients from nursing homes or rehabilitation centres
		Weekly hospital-wide VRE rectal screening limited to patients admitted for at least 48 h. In high risk departments (dialysis and ICU) all admitted patients are screened, regardless of the admission time
		Start of cleaning and disinfection (250 ppm chlorine) of the entire hospital: wards are cleaned one by one, whereby patients are temporarily transferred to other (not yet cleaned) parts of the same or other wards
		Introduction of disinfectant wipes for contact surfaces in patient rooms and general areas
		Start of mandatory plenary training sessions for all healthcare workers on general precautions and cleaning issues
		Clear division of cleaning tasks for healthcare workers and cleaning personnel
	February	After cleaning of the ward: release rooms previously occupied by VRE positive patients after cleaning and disinfection based on environmental cultures
	March (Phase II)	Audits of adherence to infection control and cleaning protocols by infection control department
		Implementing screening and isolation protocol for ‘high risk’ patients (direct contacts of VRE carriers, mostly roommates)
		Reintroducing of cleaning and disinfection (250 ppm chlorine) of the entire hospital: departments are cleaned one by one, whereby patients are temporarily transferred to other (not yet cleaned) departments
		Intensifying communication to healthcare workers and managers
	November (Phase III)	Screening for VRE carriage upon admission limited to only patients with prior hospitalisation in het ADRZ hospital in period December 1, 2015–November 14, 2016
		Monthly hospital-wide VRE rectal screening limited to patients admitted for at least 48 h. In high risk departments (dialysis and ICU) all admitted patients are screened, regardless of the admission time
2017	February (End of the outbreak)	Removing all outbreak related ‘VRE suspected’ labels in the electronic patient system
		Start hospital-wide VRE rectal screening limited to patients admitted for at least 7 days

VRE suspected patients were automatically labelled in the electronic patient system. The decision to pre-emptively isolate these patients, and the number of cultures required to remove the ‘VRE suspected’ label were adjusted in time over the course of the outbreak, and with the risk profile of the patients involved, as indicated in Table 1 and further specified in the results section below.

To detect unnoticed VRE transmission in the hospital, regular screening of all patients hospitalised for more than 48 h, and those admitted to high risk units (ICU, dialysis) was implemented during the entire outbreak (Table 1).

### Audits, cleaning and education

Cleaning tasks had to be performed by nurses or by dedicated cleaning personnel depending on the objects. During audits it became clear that the tasks had not been defined clearly and consequently some items were not always cleaned. As an intervention the tasks were specified in writing and subsequently the cleaning responsibilities were clearly defined. Also, damaged hospital equipment and furniture were repaired or replaced, and the cleaning frequency of sanitary facilities was increased. Audits on implementation of infection control measures and cleaning practises were performed, including adenosine triphosphate (ATP) measurements [7] performed by the infection prevention department. The ATP measurements were performed in patient rooms and common areas and to control cleaning after discharge (data not shown). All healthcare workers and cleaning personnel received mandatory training on standard infection control and cleaning policies. The number of alcohol-based hand rub (ABHR) dispensers was increased so ABHR was available at ‘point of care’ in all wards.

### Culturing and typing

#### Environmental sampling

To assess the extent of environmental contamination in general (surveillance) and after cleaning and disinfection of the patient rooms, a range of high touch surfaces and (medical) equipment were sampled using 10 cm × 10 cm sterile gauzes moistened with sterile saline [8]. See Additional file 1: Materials S2 for an overview of the most frequently sampled surfaces and equipment.

### Microbiology

Rectal swabs or feces was collected by nursing staff using the eSwab medium (Copan, Murrieta, USA). In total, 100 *μ*L eSwab transport medium was transferred to a brain heart infusion broth containing 4 mg/L amoxicillin. After an overnight incubation at 35–37 °C, 10 *μ*L of the broth was transferred to a Columbia colistin nalidixic acid—agar with 5% Sheep Blood and vancomycin (6 µg/mL) and grown overnight at 35–37 °C. For all suspected colonies growing on the selective media, species identification and susceptibility testing was performed using automated systems (Vitek MS and Vitek 2) (bioMérieux, Marcy l'Etoile, France) and E-test (bioMérieux, Marcy l'Etoile, France).

Resistance genes were detected using an in house *vanA*/*vanB* duplex polymerase chain reaction (Elisabeth-TweeSteden Hospital, Tilburg, The Netherlands, see Additional file 1: Materials S3).

Multilocus sequence typing (MLST) [9] and Amplified fragment Length Polymorphism (AFLP) was used to type VRE isolates [10]. During the peak of the outbreak, typing was not performed when isolates carried a *VanA* gene and patients involved shared a clear epidemiological link to avoid unnecessary costs. Hence, further typing was performed for 62 isolates of VRE*fm* positive patients (55 isolates of rectal screenings samples and 7 isolates of clinical samples) and three environmental isolates. An overview of the AFLP results is presented in Additional files 2 and 3.

## Results

### Outbreak overview

**Fig. 1:**
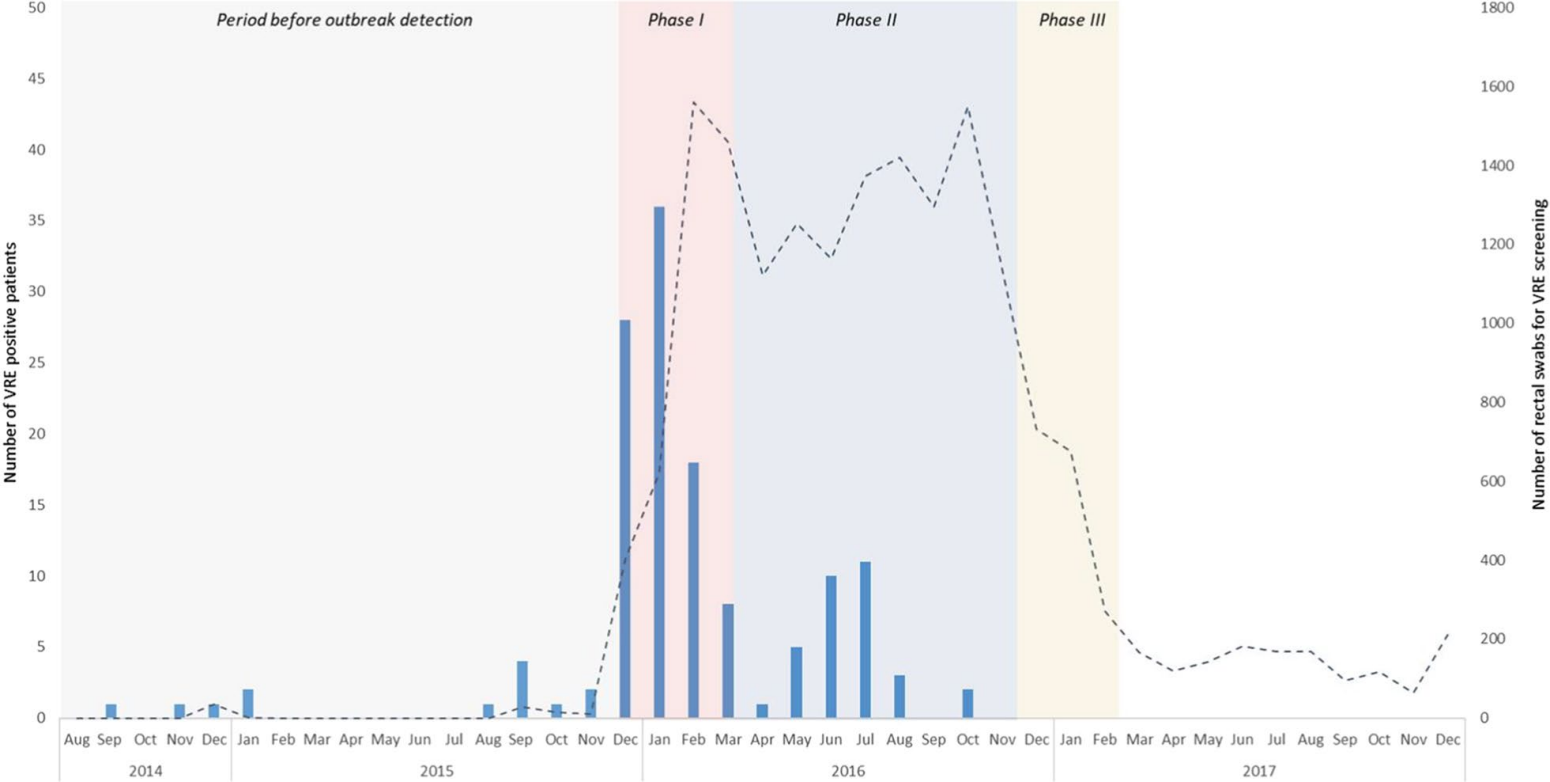
Epidemiological curve of new VRE-positive patients (blue bar, n = 135) and number of rectal screening cultures on admission, prevalence and contact tracing (dotted line) in ADRZ. Patients who were transferred to other healthcare settings when found to be VRE positive (n = 5) are not shown in this graph

From September 2014 to February 2017, a total of 140 vancomycin-resistant *Enterococcus faecium* (VRE*fm*) cases were identified. Figure 1 shows the epidemic curve of detection of new VRE*fm*-positive patients per week.

The VRE*fm* strain found during this outbreak expressed a high level of resistance to vancomycin (MIC > 256 mg/L) and teicoplanin (MIC > 32 mg/L) and carried the *vanA* gene. VRE typing of one of the first isolates showed MLST ST117, designated AFLP type AT14181; subsequent AFLP analysis of 64 isolates in the course of the outbreak period revealed a single AFLP clone (Additional file 2).

The outbreak was detected in December 2015, where VRE*fm* carriage was found in four patients who had been transferred from location Goes. On December 14, 2015, following the alert from a neighbouring academic hospital, all patients admitted for more than 48 h were screened for VRE carriage: of the 158 patients screened, 13 patients (8%) were carriers of VRE*fm*. The VRE*fm* positive patients were all admitted to various departments at location Goes (none at location Vlissingen), including the Intensive Care Unit, indicating that VRE*fm* had spread throughout the entire location (with the exception of the AAU, and the children's and maternity ward).

Retrospectively, the start of the outbreak was set at September 1, 2014, as on this date the first VRE*fm was* detected in a clinical specimen of a patient admitted to the surgical ward at location Goes. In the months following September 2014, VRE*fm* strains had been isolated in clinical materials from five patients during their admission to location Goes (Fig. 1). These had been at the time considered to be incidental findings without a clear epidemiological link. AFLP typing in December 2015 showed that these isolated belonged to a single AFLP clone (Additional file 2).

In the first phase out the outbreak from January 2016, VRE suspected patients were isolated with contact precautions (preferably in single rooms) upon (re)admission. Isolation measures were discontinued and the ‘VRE suspected’ label was removed based on a single negative rectal culture. Due to the large number of VRE suspected patients and insufficient availability of single rooms, separate cohorts were formed for VRE positive and VRE suspected patients, each with dedicated rooms, staff and (medical) equipment. Two extra five-bed-rooms were opened on the AAU to accommodate these cohorts by mid-January 2016. On the other wards, VRE suspected or positive patients were placed in a single room or cohorted with other (suspected) VRE carriers if there was insufficient availability of single rooms.

Screening of VRE suspected patients upon admission between January and March 2016 (phase I), showed that only 2 of 647 patients (0.31%) were positive for VRE*fm*. None of these patients were residing in a nursing home or rehabilitation facility. Given the low VRE prevalence in the cohort of patients admitted between September 2014 and December 2015 and the absence of VRE carriage among patients transferred from other institutions, patients from this cohort were labelled ‘medium risk’ and no longer pre-emptively isolated upon admission starting from March 21, 2016 (phase II). Screening of this cohort by performing a single rectal culture upon admission remained unchanged.

In this second phase of the outbreak, patients who had been in contact with a VRE*fm*-positive patient (roommates of VRE carriers) were labelled ‘high risk’, isolated and screened using 3 separate rectum cultures on day 3, 5 and 7 after last exposure (Table 1).

After an initial decline in the number of new VRE*fm* findings in February and March, 2016—there was a second peak in the number of VRE*fm* positive patients in May–June, 2016. On-site audits performed during phase II showed shortcomings in infection control preconditions on several wards in the hospital: wards were cluttered, the separation of dirty and clean areas was not clear, and the replacement of damaged hospital equipment and furniture had not been implemented. The division of labour with regards to cleaning responsibilities between cleaning personnel and healthcare workers was further emphasized in this phase, and environmental sampling increased in frequency (see below).

As of November 2016, there were no new VRE*fm* findings in the preceding three months and therefore screening for VRE carriage on admission was limited to only patients with prior hospitalisation in het ADRZ hospital in the period December, 2015–November, 2016 (phase III). Furthermore, the frequency of hospital-wide screening of patients with > 48 h length of stay was from then on performed monthly instead of weekly.

### Control of the outbreak

No further cases occurred over a three months period and control measures were terminated in February, 2017. Admission and prevalence surveillance cultures were discontinued and all outbreak related ‘VRE suspected’ labels in the electronic patient system were removed. Furthermore, a hospital-wide VRE rectal screening limited to patients admitted for at least 7 days, was implemented as a standard surveillance form that moment on.

### Environmental cultures

**Fig. 2:**
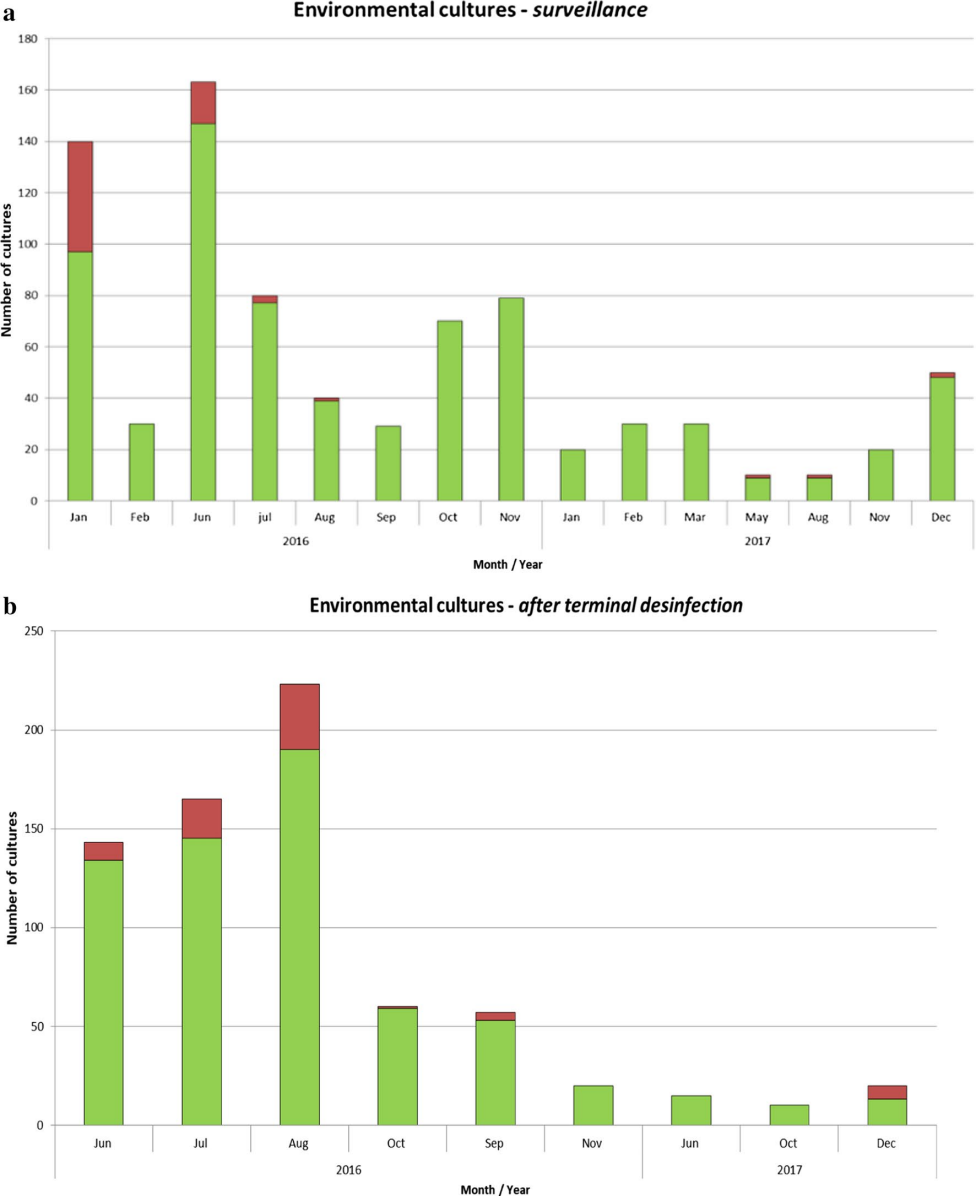
Environmental cultures of the patient rooms, a range of high touch surfaces and (medical) equipment’s at random times (surveillance) (**a**) and after disinfection (**b**). Red bar: VREfm positive cultures; green bar: VRE negative cultures

In January 2016, environmental cultures were obtained throughout the hospital to assess the extent of environmental contamination. The cultures showed extensive VRE contamination on the surgical, internal, pulmonary and neurology wards (43/80 samples VRE positive; 53.7%). Environmental samples of the AUU, ICU and dialysis department were VRE negative (0/60 samples). (Fig. 2a).

In June 2016, environmental screening was repeated on multiple wards (n = 130 samples), and again extensive VRE contamination was found in the surgical ward (19/20 samples, 95.0%) and to a lesser extent on the cardiology ward (4/20 samples; 20.0%). Consequently, stepwise cleaning and disinfection (250 ppm chlorine) of these wards was performed. After cleaning these wards were closed pending VRE negative environmental results. Following a peak in VRE transmission, environmental surveillance was continued and intensified: from June 2016, rooms previously occupied by VRE-positive patients were only released after cleaning and negative environmental cultures. Ten percent (74/713 culture) of the room surfaces remained VRE*fm* positive after terminal disinfection (Fig. 2b). In some cases, VRE*fm* was still detected after two rounds of terminal disinfection on e.g. patient bed, infusion pole and pull-up bar.

### Infections during the outbreak period

Eight (5.7%) patients developed a *VREfm* infection, of whom two (1.4%) had bacteraemia. Two patients, with extensive co-morbidities, died shortly after detection. One patient, also with extensive co-morbidities (including renal failure, haemodialysis and vascular disease) developed a severe osteomyelitis following a surgical procedure, which eventually led to amputation of her left hand.

## Discussion

Here we describe the successful control of a VRE*fm* outbreak in a hospital using a more restrictive screening and isolation policy than recommended in the national guidelines [6]. With this approach, within nine months after the detection of the outbreak, no new VRE*fm* cases were detected and after twelve months the outbreak was considered fully controlled. In addition to the targeted screening and isolation there was an intensive focus on optimisation of environmental cleaning procedures.

In general, there is no consensus on the optimal VRE screening, isolation and surveillance protocol, reflected by the variation in infection control approaches within and between countries [11–13]. The number of rectal cultures required to consider a known carrier or contact patient VRE-negative, is unclear. Studies show that the sensitivity of a single rectal swab is low, ranging from 42.5 to 79% [14–17], and this increases when taking multiple swabs: Pearman et al*.* showed that on average four rectal swabs, collected on separate days, were needed to detect 95% of carriers compared to 56% with one rectal swab [15]. Explanations for the increase in sensitivity when taking multiple rectal swabs include a fluctuation in faecal excretion of VRE, and/or the presence of an intestinal transit time after VRE is transmitted (time between transmission event and detectable VRE levels in the faeces). It should be noted that in most of these studies the rectal swabs were inoculated directly on selective media. Addition of a broth enrichment step (as done in our study) increases the yield of a rectal swab culture substantially [18]. Lastly, sensitivity may depend on the load of VRE in faeces [17]; a high VRE load in faeces also results in a higher sensitivity of a rectal swab, whereby patients with lower faecal loads probably contribute less to transmission.

In 2015, a Dutch guideline was published which recommends taking 3–5 rectal cultures on separate days to reliably exclude carriage in a suspected VRE carrier [6]. This guideline makes no specific recommendation about the timing of the 3–5 separate rectal culture collection, apart from recommending that the last culture is ideally performed at least seven days after the last exposure. During our outbreak, the screening policy consisting of a single rectal swab culture (with enrichment broth) upon re-admission for excluding VRE carriage in the majority of VRE suspected patients in the first phase of the outbreak, and a maximum of three culture in the second phase for ‘high risk’ VRE suspected patients.

In this outbreak, there was a long period of time between the first clinical VRE finding (September 2014) and the detection of the outbreak (December 2015), resulting in a large cohort of approximately 25,000 discharged and potentially exposed patients, hence classified as a ‘VRE suspected’. Readmission of these patients occurred frequently and it was estimated, based on historical data, that approximately 4700 admissions/year would be categorised as ‘VRE suspected’. According to the guideline this would result in 75,000–125,000 cultures to detect all carriers (by screening all discharged patients). This would have required considerable administrative effort and laboratory costs. In case of limiting the screening to those who were re-admitted (appr. 4700/year), isolating all ‘VRE suspected’ patients in a single room upon admission pending at least 3 rectal screening cultures on separate days would place a large burden on hospital room capacity, and still require a very large number of cultures.

With the assumption that the sensitivity of a single swab is substantially higher when performed > 7 days after the last exposure, and because a substantial proportion of exposed patients was expected to have lost VRE carriage within 6 months [19], we decided to perform screening for VRE by taking a single swab, and limit screening to patients who were re-admitted to the hospital. By ending pre-emptive contact precautions after a single VRE-negative rectal swab, most patients were isolated only the first two days of admission. With this strategy, that was less stringent than the Dutch national recommendations for VRE control, a total of 19,677 rectal swabs (of 9279 patients) were collected during the entire outbreak (admission-, prevalence- and contact surveillance cultures together), thereby significantly cutting administrative effort, time of isolation and laboratory costs.

Though it can be argued that we have not detected all VRE*fm* carriers (and underestimated the size of the outbreak) due to suboptimal sensitivity of our screening policy, our experience shows that VRE transmission from undetected carriers was, even if present, insufficient to caused sustained transmission. Whether this strategy would have been effective in settings with higher complexity of care (where patients probably have longer duration of carriage, longer admissions, and a higher transmission potential and infection risk) is unclear. In such settings, even a small loss in sensitivity may lead to ongoing transmission. In a recent paper, Frakking et al*.* describe a successful control of a large VRE outbreak (n = 242 patients) in a Dutch teaching hospital and tertiary referral centre [4]. The outbreak lasted 18 months and was eventually controlled after major efforts, including twice-weekly screening of all admitted patients, environmental decontamination with hydrogen peroxide vapour, strict isolation precautions (in isolation rooms with anteroom), a VRE quarantine ward and abandoning the use of ciprofloxacin prophylaxis during neutropenic fever. The authors advocate a screening policy with at least 4–5 rectal swabs before excluding VRE carriage.

It should be noted that in our study, for the majority of the cohort of suspected VRE patients the last exposure was > 7 days ago (for many patients even several months), which (in combination with intensive focus on general precautions and environmental cleaning) may have been an important reason for the success of this strategy. In addition, the prevalence among re-admitted patients was < 1%, indicating a low background risk of undetected introductions of VRE in the hospital.

As described we are careful to generalize our findings to other settings. Our results suggest, however, that the current general Dutch recommendations to take 3–5 cultures to exclude VRE carriage in all exposed patients may be reconsidered for centres with lower complexity of care, especially when the last exposure was > 7 days ago. This lowers the costs and limits the duration of pre-emptive isolation.

## Conclusion

To conclude, we describe a large VRE outbreak in a general hospital in The Netherlands, that was successfully controlled, while substantially reducing the number of cultures to be taken and the number of isolation days, and thereby cutting laboratory costs.

## Supplementary Information

**Additional file 1**. Additional files.

**Additional file 2**. Dendrogram based on Amplified fragment length polymorphism (AFLP) of 65 VRE strains identified during the outbreak. E. faecium (CDC NY2) and E. faecalis (ATCC 29212) were included in the analysis as reference strains. The cut-off value for identical strains was 90% relative similarity.

**Additional file 3**. Dendrogram based on Amplified fragment length polymorphism (AFLP) of 12 VRE strains identified between 2016 and 2020 in ADRZ - NOT belonging to the outbreak cluster. E. faecium (CDC NY2) and E. faecalis (ATCC 29212) were included in the analysis as reference strains. The cut-off value for identical strains was 90% relative similarity.

## Data Availability

The datasets used and/or analysed during the current study are available from the corresponding author on reasonable request.

## Abbreviations

AAU: Acute admission unit
ADRZ: Admiraal De Ruyter Hospital (ADRZ)
AFLP: Amplified fragment length polymorphism
ICU: Intensive care unit
MLST: Multilocus sequence typing
MRSA: Methicillin-resistant *Staphylococcus aureus*
VRE: Vancomycin-resistant enterococci
VRE*fm*: Vancomycin-resistant *Enterococcus faecium*
