# Supplementary material for: Management of a hospital-wide vancomycin-resistant Enterococcus faecium outbreak in a Dutch general hospital, 2014–2017: successful control using a restrictive screening strategy
Source: Antimicrob Resist Infect Control. 2021 Feb 18;10:38. doi: 10.1186/s13756-021-00906-x (PMC7893727; doi:10.1186/s13756-021-00906-x)
Supplement: Supplementary file 1 — Additional file 1. Additional files. [file 13756_2021_906_MOESM1_ESM.docx]

**Supplementary material**

**S1. Number of new cases of VRE bacteraemia and VRE bacteraemia per 100.000 patient days in The Admiraal De Ruyter Hospital, 2014 - 2020**

|  | **2014** | **2015** | **2016** | **2017** | **2018** | **2019** | **2020*** |
| --- | --- | --- | --- | --- | --- | --- | --- |
| *Number of new cases of VRE bacteraemia* | 0 | 2 | 0 | 0 | 0 | 0 | 0 |
| *VRE bacteraemia per 100.000 patient days* | 0 | 4,4 | 0 | 0 | 0 | 0 | 0 |

***** *only data until December, 28, 2020 were included*

**S2. Overview of the items sampled during the outbreak, indicating which items were tested VRE positive at least once.**

| **Item** | **Tested VRE positive at least once** |
| --- | --- |
| *Patient room* | |
| Hospital bed | **Yes** |
| Pillow | **Yes** |
| Mattress | **Yes** |
| Pull-up bracket | **Yes** |
| Paging system at bed | No |
| Nightstand | **Yes** |
| Telephone patient room | **Yes** |
| Electricity bar in patient room | **Yes** |
| Sink | No |
| Shower | **Yes** |
| Toilet | **Yes** |
| Doorknob bathroom | No |
| Chair | **Yes** |
| Table | No |
| *Medical devices* | |
| Glucose meter | No |
| Blood pressure meter | **Yes** |
| Ear thermometer | No |
| Stethoscope | No |
| Infusion pomp | No |
| *Ward bounds materials* | |
| Computer in teampost | No |
| Telephone teampost | No |
| Paper medical records | **Yes** |
| Door handles sterile storage | No |
| Pneumatic tube system | **Yes** |
| Computer on wheels | **Yes** |
| Commode chair | **Yes** |
| Corridor wall hand rail | **Yes** |
| Walker | No |
| storage trays | No |
| Others | No |

**S3. Protocol of the in house *vanA*/*vanB* duplex polymerase chain reaction performed at the Elisabeth-TweeSteden Hospital, Tilburg, The Netherlands**

DNA was extracted using the QIAsymphony DSP virus/pathogen midi kit and pathogen complex 400 protocol of the QIAsymphony Sample Processing (SP) system (Qiagen, Hilden, Germany). Amplification reactions were performed in a volume of 25 *μ*L with PCR mastermix (QuantiTect Multiplex PCR NoROX Kit, QIAgen) and 10 *μ*L DNA sample. A multiplex PCR using *vanA*-, *vanB*-, and *E. faecium*-specific primers and probes (**table A**) was performed. For the amplification and detection of the internal control PCR primers and detection probe specific for PhHV-1 were used (Niesters HGM. Clinical virology in real time. J Clin Virol Off Publ Pan Am Soc Clin Virol. 2002 Dec;25 Suppl 3:S3-12.). The amplification reaction was performed using 200 nM of each *vanA*, *vanB* and *E. faecium*-specific primers, 100 nM of each PhHV-1-specific primer, and 100 nM of each *vanA*-, *vanB*-, *E. faecium*- PhHV-specific probe.

Amplification consisted of 15 minutes at 95°C followed by 45 cycles of 15 seconds at 95°C, 30 seconds at 60°C, and 15 seconds at 72°C. Amplification, detection, and analysis were performed with the Rotor-gene real-time detection system (QIAgen). Negative and positive control samples were included in each amplification run. PCR output from this system consists of a Ct-value, representing the amplification cycle in which the level of fluorescent signal exceeds the background fluorescence, and reflecting the target-specific DNA load in the sample tested.

***Table A*** *Primer and sequences for multiplex PCR of vancomycin resistance genotyping and enterococci species identification among vancomycin-resistant enterococci*

| **Primer name** | **Primer sequence** | **Target gene** |
| --- | --- | --- |
| **vanA-F1** | GCCGGAAAAAGGCTCTGAA | *vanA gene* |
| **vanA-R1** | TCCTCGCTCCTCTGCTGAA | *vanA gene* |
| **vanA-1-FAMBhq1** | ACGCAGTTATAACCGTTCCCGCAGACC | *vanA gene* |
| **vanB-F1** | CGCAGCTTGCATGGACAA | *vanB gene* |
| **vanB-R1** | GGCGATGCCCGCATT | *vanB gene* |
| **vanB-1-VIC** **(MGB)** | TCACTGGCCTACATTC | *vanB gene* |
| **Efa-F1** | GGAATGGCGCAAAACTTAGA | *atpA gene* |
| **Efa-R1** | AGGCCTCTCCAACTGGAACT | *atpA gene* |
| **Efa-1-TRBhq2** | TGGCGATTTCGAGTCCATTCG | *atpA gene* |
