# Supplementary figures and images for: Management of a hospital-wide vancomycin-resistant Enterococcus faecium outbreak in a Dutch general hospital, 2014–2017: successful control using a restrictive screening strategy
Source: Antimicrob Resist Infect Control. 2021 Feb 18;10:38. doi: 10.1186/s13756-021-00906-x (PMC7893727; doi:10.1186/s13756-021-00906-x)

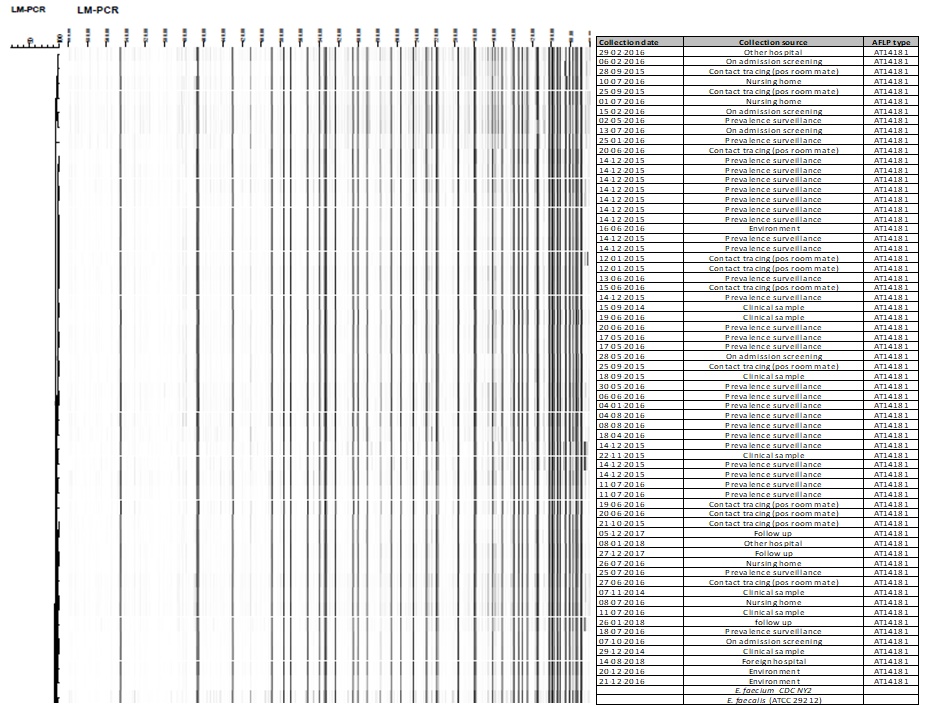

Supplement: Supplementary file 2 — Additional file 2. Dendrogram based on Amplified fragment length polymorphism (AFLP) of 65 VRE strains identified during the outbreak. E. faecium (CDC NY2) and E. faecalis (ATCC 29212) were included in the analysis as reference strains. The cut-off value for identical strains was 90% relative similarity. [file 13756_2021_906_MOESM2_ESM.jpg]

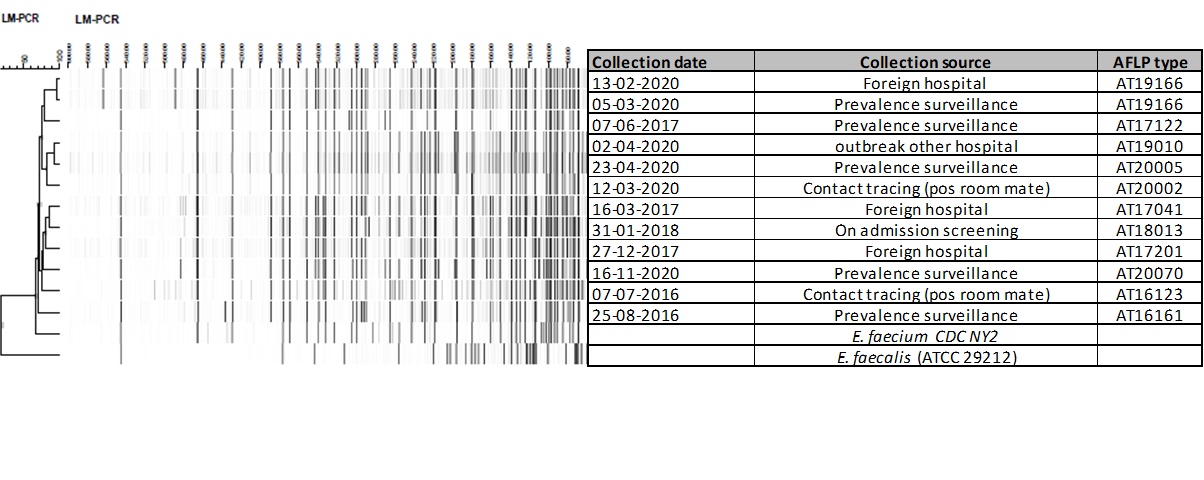

Supplement: Supplementary file 3 — Additional file 3. Dendrogram based on Amplified fragment length polymorphism (AFLP) of 12 VRE strains identified between 2016 and 2020 in ADRZ - NOT belonging to the outbreak cluster. E. faecium (CDC NY2) and E. faecalis (ATCC 29212) were included in the analysis as reference strains. The cut-off value for identical strains was 90% relative similarity. [file 13756_2021_906_MOESM3_ESM.jpg]
